# Supplementary material for: The Invasive Tradescantia zebrina Affects Litter Decomposition, but It Does Not Change the Lignocellulolytic Fungal Community in the Atlantic Forest, Brazil
Source: Plants (Basel). 2023 May 30;12(11):2162. doi: 10.3390/plants12112162 (PMC10255722; doi:10.3390/plants12112162)
Supplement: Supplementary file 1 [file plants-12-02162-s001.zip › plants-2267616-supplementary.pdf]

**Table S1.** Comparison of the sequence from isolates with sequences deposited in databases ("blast").

| Isolated | | % Similarity | E Value | Identification | Access number | Final Identification |
|---|---|---|---|---|---|---|
| **1** | **2AO** | 99.76 | 0.0 | Fusarium camptoceras | KJ782411.1 | Fusarium sp. |
| | | 99.76 | 0.0 | Fusarium equiseti | AB425996.1 | |
| | | 99.76 | 0.0 | Fusarium oxysporum | JN400714.1 | |
| | | 99.76 | 0.0 | Fusarium sp. | HQ023205.1 | |
| | | 99.52 | 0.0 | Fusarium equiseti | KP026922.1 | |
| **2** | **2AQ** | 99.19 | 0.0 | Trametes polyzona | JN164979.1 | Trametes sp. |
| | | 98.49 | 0.0 | Trametes polyzona | MH855813.1 | |
| | | 98.48 | 0.0 | Trametes polyzona | KT120039.1 | |
| | | 98.39 | 0.0 | Trametes polyzona | JN164978.1 | |
| **3** | **2AN** | 97.19 | 0.0 | Fusarium sp. | HQ023200.1 | Fusarium sp. |
| | | 96.68 | 0.0 | Fusarium sp. | U85548.1 | |
| | | 95.62 | 0.0 | Fusarium robustum | NR_159851.1 | |
| | | 95.62 | 0.0 | Fusarium robustum | MH861018.1 | |
| | | 95.56 | 0.0 | Fusarium sp. | HQ023198.1 | |
| **4** | **2BD** | 99.39 | 0.0 | Fusarium equiseti | KP026922.1 | Fusarium sp. |
| | | 99.59 | 0.0 | Fusarium equiseti | AB425996.1 | |
| | | 99.59 | 0.0 | Fusarium oxysporum | JN400714.1 | |
| | | 99.38 | 0.0 | Fusarium equiseti | GQ365157.1 | |
| **5** | **2M** | 98.41 | 0.0 | Aspergillus caelatus | MH862672.1 | Aspergillus sp. |
| | | 98.27 | 0.0 | Aspergillus caelatus | KM613140.1 | |
| | | 98.12 | 0.0 | Aspergillus pseudota-marii | MH862673.1 | |
| | | 98.12 | 0.0 | Aspergillus pseudota-marii | DQ467987.1 | |
| | | 98.12 | 0.0 | Aspergillus pseudota-marii | DQ467986.1 | |
| **6** | **2S** | 99.79 | 0.0 | Phoma sp. | KP966098.1 | Phoma sp. |
| | | 99.79 | 0.0 | Epicoccum sorghinum | MN215627.1 | |
| | | 99.79 | 0.0 | Epicoccum sorghinum | MN215624.1 | |
| | | 99.79 | 0.0 | Epicoccum sorghinum | MN215623.1 | |
| | | 99.79 | 0.0 | Epicoccum sorghinum | MN215622.1 | |
| **7** | **2AM** | 100.00 | 0.0 | Alternaria doliconidium | NR_158361.1 | Alternaria sp. |
| | | 100.00 | 0.0 | Alternaria doliconidium | MG828864.1 | |
| | | 100.00 | 0.0 | Alternaria alternata | LN808867.1 | |
| | | 100.00 | 0.0 | Alternaria sp. | HQ649963.1 | |
| | | 99.81 | 0.0 | Alternaria sp. | MG209668.1 | |

| | | | | | | |
|---|---|---|---|---|---|---|
| **8** | **2E** | 99.79 | 0.0 | Aspergillus sydowii | KP973600.1 | Aspergillus sp. |
| | | 99.37 | 0.0 | Aspergillus sydowii | LN898721.1 | |
| | | 99.37 | 0.0 | Aspergillus sydowii | LN898720.1 | |
| | | 99.37 | 0.0 | Aspergillus sydowii | LN898719.1 | |
| | | 99.37 | 0.0 | Aspergillus sydowii | LN898716.1 | |
| **9** | **2AJ** | 99.57 | 0.0 | Fusarium oxysporum | MG356946.1 | Fusarium sp. |
| | | 99.57 | 0.0 | Fusarium oxysporum | KU382653.1 | |
| | | 99.57 | 0.0 | Fusarium sp. | GQ365161.1 | |
| | | 99.57 | 0.0 | Fusarium oxysporum | GQ365156.1 | |
| | | 99.57 | 0.0 | Fusarium sp. | EU363511.1 | |
| **10** | **1BH** | 99.79 | 0.0 | Gibberella moniliformis | EU151467.1 | Fusarium sp. |
| | | 99.79 | 0.0 | Gibberella fujikuroi | AY188916.1 | |
| | | 99.79 | 0.0 | Melanospora zamiae | KY628680.1 | |
| | | 99.58 | 0.0 | Gibberella moniliformis | EU151476.1 | |
| | | 99.58 | 0.0 | Fusarium denticulatum | U61680.1 | |
| **11** | **2BH** | 99.42 | 0.0 | Bipolaris gossypina | MH864410.1 | Bipolaris sp. |
| | | 99.02 | 0.0 | Bipolaris sorokiniana | KR812216.1 | |
| | | 98.65 | 0.0 | Curvularia sp. | KR093890.1 | |
| | | 98.23 | 0.0 | Bipolaris salviniae | MH864690.1 | |
| | | 98.23 | 0.0 | Bipolaris salviniae | MH864687.1 | |
| **12** | **2L** | 99.78 | 0.0 | Curvularia sp. | MG976352.1 | Curvularia sp. |
| | | 99.78 | 0.0 | Curvularia soli | NR_152503.1 | |
| | | 99.78 | 0.0 | Curvularia soli | KY905679.1 | |
| | | 99.78 | 0.0 | Curvularia sp. | KT777714.1 | |
| | | 99.78 | 0.0 | Curvularia sp. | KR093913.1 | |
| **13** | **2BB** | 100.00 | 0.0 | Aspergillus sp. | KU751784.1 | Aspergillus sp. |
| | | 92.11 | 2e-151 | Aspergillus indologenus | MH861245.1 | |
| | | 92.11 | 2e-151 | Aspergillus aculeatus | AY585557.1 | |
| | | 91.71 | 2e-151 | Aspergillus uvarum | MH863120.1 | |
| **14** | **1AI** | 99.77 | 0.0 | Colletotrichum acidae | MG996506.1 | Colleototrichum sp. |
| | | 99.77 | 0.0 | Colletotrichum acidae | NR_160841.1 | |
| | | 97.81 | 0.0 | Colletotrichum capsici | EU056738.1 | |
| **15** | **2AL** | 99.37 | 0.0 | Fusarium oxysporum | MG356946.1 | Fusarium sp. |
| | | 99.37 | 0.0 | Fusarium oxysporum | KU382653.1 | |
| | | 99.37 | 0.0 | Fusarium sp. | GQ365161.1 | |
| | | 99.37 | 0.0 | Fusarium oxysporum | GQ365156.1 | |
| | | 99.37 | 0.0 | Fusarium sp. | EU363511.1 | |

| | | | | | | |
|---|---|---|---|---|---|---|
| **16** | **2AH** | 99.79 | 0.0 | Fusarium chlamydo-sporum | KM076600.1 | Fusarium sp. |
| | | 99.58 | 0.0 | Fusarium equiseti | AY213655.1 | |
| | | 99.37 | 0.0 | Fusarium sp. | MH582470.1 | |
| | | 99.37 | 0.0 | Fusarium sp. | MH582467.1 | |
| | | 99.37 | 0.0 | Fusarium sp. | MH582450.1 | |
| **17** | **1L** | 99.61 | 0.0 | Aspergillus giganteus | MH857126.1 | Aspergillus sp. |
| | | 99.59 | 0.0 | Aspergillus giganteus | KR296897.1 | |
| | | 99.59 | 0.0 | Aspergillus giganteus | NR_135403.1 | |
| | | 99.59 | 0.0 | Aspergillus giganteus | EF669987.1 | |
| | | 99.59 | 0.0 | Aspergillus giganteus | EF669928.1 | |
| **18** | **2F** | 99.59 | 0.0 | Aspergillus fumigatus | MH864623.1 | Aspergillus sp. |
| | | 99.59 | 0.0 | Aspergillus fumigatus | MF476024.1 | |
| | | 99.59 | 0.0 | Aspergillus fumigatus | KJ863511.1 | |
| | | 99.59 | 0.0 | Aspergillus fumigatus | KP724993.1 | |
| | | 99.59 | 0.0 | Aspergillus fumigatus | KP724992.1 | |
| **19** | **2AC** | 99.00 | 0.0 | Fusarium subglutinans | EU117221.1 | Fusarium sp. |
| | | 99.00 | 0.0 | Fusarium proliferatum | EF589878.1 | |
| | | 99.00 | 0.0 | Fusarium sp. | KM104592.1 | |
| **20** | **2AU** | 99.58 | 0.0 | Colletotrichum fusiforme | NR_160841.1 | Colletotrichum sp. |
| | | 99.38 | 0.0 | Colletotrichum acidae | MG996506.1 | |
| | | 97.62 | 0.0 | Colletotrichum capsici | EU056738.1 | |
| | | 97.43 | 0.0 | Colletotrichum capsici | LC152972.1 | |
| | | 97.43 | 0.0 | Glomerella truncata | FJ172230.1 | |
| **21** | **2AR** | 98.96 | 0.0 | Colletotrichum acidae | NR_160841.1 | Colletotrichum sp. |
| | | 98.76 | 0.0 | Colletotrichum acidae | MG996506.1 | |
| | | 97.24 | 0.0 | Colletotrichum capsici | EU056738.1 | |
| | | 97.05 | 0.0 | Colletotrichum capsici | LC152972.1 | |
| **22** | **1AN** | 98.39 | 0.0 | Fusarium incarnatum | MF356578.1 | Fusarium sp. |
| | | 98.20 | 0.0 | Fusarium chlamydo-sporum | KM076600.1 | |
| | | 98.20 | 0.0 | Fusarium sp. | GQ352485.1 | |
| | | 98.20 | 0.0 | Fusarium incarnatum | MH865893.1 | |
| **23** | **1BL** | 99.56 | 0.0 | Fusarium proliferatum | MG228402.1 | Fusarium sp. |
| | | 99.56 | 0.0 | Fusarium proliferatum | MG228401.1 | |
| | | 99.56 | 0.0 | Fusarium sp. | MK432970.1 | |
| | | 99.13 | 0.0 | fusarium oxysporum | JN400715.1 | |
| | | 99.13 | 0.0 | fusarium oxysporum | JN400698.1 | |

| | | | | | | |
|---|---|---|---|---|---|---|
| **24** | **1K** | 99.16 | 0.0 | Penicillium citrinum | KM613148.1 | Penicillium sp. |
| | | 98.57 | 0.0 | Penicillium citrinum | KX066022.1 | |
| | | 98.57 | 0.0 | Penicillium citrinum | KX066021.1 | |
| | | 98.37 | 0.0 | Penicillium sp. | GU017521.1 | |
| | | 98.37 | 0.0 | Penicillium griseofulvum | FJ717687.1 | |
| **25** | **2AS** | 99.58 | 0.0 | Volutella lini | JQ693169.1 | Volitella sp. |
| | | 99.41 | 0.0 | Volutella lini | JQ647452.1 | |
| | | 93.35 | 0.0 | volutella consors | KM231768.1 | |
| **26** | **2AV** | 98.96 | 0.0 | Fusarium sp. | HQ023200.1 | Fusarium sp. |
| | | 98.16 | 0.0 | Fusarium robustum | NR_159851.1 | |
| | | 98.16 | 0.0 | Fusarium robustum | MH861018.1 | |
| | | 97.96 | 0.0 | Fusarium robustum | U85539.1 | |
| | | 97.69 | 0.0 | Gibberella pulicaris | DQ026011.1 | |
| **27** | **2AZ** | 93.33 | 0.0 | Gibberella moniliformis | EU151482.1 | NI |
| | | 93.26 | 0.0 | Fusarium sp. | KU382552.1 | |
| **28** | **2BA** | 98.13 | 0.0 | Fusarium equiseti | HQ649909.1 | Fusarium sp. |
| | | 98.13 | 0.0 | Fusarium equiseti | HQ649904.1 | |
| | | 98.13 | 0.0 | Fusarium equiseti | HQ649906.1 | |
| | | 98.13 | 0.0 | Fusarium equiseti | HQ649905.1 | |
| | | 97.93 | 0.0 | Fusarium caatingaense | MH668833.1 | |
| **29** | **2BF** | 98.38 | 0.0 | Fusarium sp. | GQ352485.1 | Fusarium sp. |
| | | 98.38 | 0.0 | Fusarium chlamydo-sporum | MF072670.1 | |
| | | 98.38 | 0.0 | Fusarium incarnatum | MF356578.1 | |
| **30** | **2N** | 98.25 | 0.0 | Curvularia sp. | HQ909079.1 | Curvularia sp. |
| | | 98.24 | 0.0 | Curvularia clavata | MN592977.1 | |
| | | 98.24 | 0.0 | Curvularia sp. | MG976352.1 | |
| | | 98.24 | 0.0 | Curvularia soli | NR_152503.1 | |
| | | 98.24 | 0.0 | Curvularia soli | KY905679.1 | |
| **31** | **2Y** | 94.91 | 0.0 | Byssochlamys spectabilis | MH856887.1 | NI |
| | | 94.91 | 0.0 | Paecilomyces variotii | JF766675.1 | |
| | | 94.91 | 0.0 | Paecilomyces variotii | GU968674.1 | |
| | | 94.91 | 0.0 | Paecilomyces variotii | FJ389930.1 | |
| | | 94.91 | 0.0 | Paecilomyces variotii | AF033395.1 | |
| **32** | **2O** | 98.90 | 0.0 | Dothideales sp. | HQ608008.1 | Epicoccum sp. |
| | | 98.90 | 0.0 | Epicoccum sp. | MG976442.1 | |
| | | 98.90 | 0.0 | Epicoccum sp. | MG976431.1 | |
| | | 98.90 | 0.0 | Epicoccum sp. | LT592959.1 | |

| | | | | | | |
|---|---|---|---|---|---|---|
| | | 98.90 | 0.0 | Epicoccum sp. | LT592927.1 | |
| **33** | **2R** | 95.63 | 0.0 | Montagnula cirsii | KX274242.1 | Montagnula sp. |
| | | 95.49 | 0.0 | Montagnula scabiosae | NR_155378 | |
| | | 95.49 | 0.0 | Montagnula scabiosae | KT443907 | |
| **34** | **2B** | 99.29 | 0.0 | Trichoderma hamatum | MH864073.1 | Trichoderma sp. |
| | | 99.12 | 0.0 | Trichoderma harzianum | KR868288.1 | |
| | | 99.12 | 0.0 | Hypocrea lixii | HQ011501.1 | |
| | | 99.12 | 0.0 | Trichoderma harzianum | KR868336.1 | |
| | | 99.12 | 0.0 | Trichoderma harzianum | KR868321.1 | |
| **35** | **2J** | 99.08 | 0.0 | Fusarium oxysporum | DQ068983.1 | Fusarium sp. |
| | | 98.87 | 0.0 | Fusarium oxysporum | KX786247.1 | |
| | | 98.87 | 0.0 | Fusarium oxysporum | KX786244.1 | |
| | | 98.87 | 0.0 | Fusarium oxysporum | KX786241.1 | |
| | | 98.87 | 0.0 | Fusarium oxysporum | LN828174.1 | |
| **36** | **1AS** | 99.31 | 0.0 | Fusarium solani | MH348949.1 | Fusarium sp. |
| | | 99.31 | 0.0 | Fusarium solani | MH348946.1 | |
| | | 99.31 | 0.0 | Fusarium solani | MH348944.1 | |
| | | 99.30 | 0.0 | Fusarium solani | LN828130.1 | |
| | | 98.85 | 0.0 | Fusarium solani | MH865999.1 | |
| **37** | **2AW** | 98.88 | 0.0 | Clonostachys sp. | KY413708.1 | Clonostachys sp. |
| | | 98.88 | 0.0 | Clonostachys sp. | KY413698.1 | |
| | | 98.62 | 0.0 | Gliocladium sp. | AF139857.1 | |
| | | 98.42 | 0.0 | Clonostachys sp. | MH267864.1 | |
| | | 98.42 | 0.0 | Clonostachys rogersoniana | KR812215.1 | |
| **38** | **2A** | 98.99 | 0.0 | Cladosporium cladosporioides | MF044039.1 | Cladosporium sp. |
| | | 98.99 | 0.0 | Cladosporium tenuissimum | MF473296.1 | |
| | | 98.99 | 0.0 | Cladosporium tenuissimum | MF473282.1 | |
| | | 98.99 | 0.0 | Cladosporium tenuissimum | MF473290.1 | |
| | | 98.99 | 0.0 | Cladosporium tenuissimum | LN834398.1 | |
| **39** | **1G** | 99.77 | 0.0 | Aspergillus sydowii | MH858253.1 | Aspergillus sp. |
| | | 99.77 | 0.0 | Aspergillus sydowii | MH854859.1 | |
| | | 99.77 | 0.0 | Aspergillus sp. | MG976613.1 | |
| | | 99.77 | 0.0 | Aspergillus sydowii | LN898735.1 | |
| | | 99.77 | 0.0 | Aspergillus sydowii | LN898734.1 | |

| | | | | | | |
|---|---|---|---|---|---|---|
| **40** | **1M** | 99.60 | 0.0 | Aspergillus sydowii | MH854859.1 | Aspergillus sp. |
| | | 99.60 | 0.0 | Aspergillus sp. | MG976613.1 | |
| | | 99.60 | 0.0 | Aspergillus sydowii | LN898735.1 | |
| | | 99.60 | 0.0 | Aspergillus sydowii | LN898734.1 | |
| | | 99.60 | 0.0 | Aspergillus sydowii | KT826624.1 | |
| **41** | **1J** | 96.30 | 0.0 | Cladosporium cladospori-oides | MF072635.1 | Cladosporium sp. |
| | | 96.30 | 0.0 | Cladosporium cladospori-oides | MF072642.1 | |
| | | 96.30 | 0.0 | Cladosporium sp. | LN809014.1 | |
| | | 96.30 | 0.0 | Cladosporium cladospori-oides | LN809009.1 | |
| | | 96.30 | 0.0 | Cladosporium cladospori-oides | LN809000.1 | |
| **42** | **1AE** | 99.22 | 0.0 | Aspergillus versicolor | AM883155.1 | Aspergillus sp. |
| | | 99.04 | 0.0 | Aspergillus tabacinus | LN898739.1 | |
| | | 99.04 | 0.0 | Aspergillus tabacinus | LN898738.1 | |
| | | 99.04 | 0.0 | Aspergillus tabacinus | LN898737.1 | |
| | | 99.04 | 0.0 | Aspergillus tabacinus | LN898736.1 | |
| **43** | **1AM** | 98.23 | 0.0 | Fusarium chlamydo-sporum | AY213655.1 | Fusarium sp. |
| | | 98.01 | 0.0 | Fusarium chlamydo-sporum | KM076600.1 | |
| | | 97.79 | 0.0 | Fusarium incarnatum | MH865897.1 | |
| | | 97.79 | 0.0 | Fusarium incarnatum | MH865895.1 | |
| | | 97.79 | 0.0 | Fusarium camptoceras | MH858537.1 | |
| **44** | **2AA** | 97.94 | 0.0 | Trichoderma spirale | FJ861463.1 | Trichoderma sp. |
| | | 97.38 | 0.0 | Trichoderma harzianum | FJ884153.1 | |
| | | 97.37 | 0.0 | Trichoderma spirale | KR296909.1 | |
| | | 97.22 | 0.0 | Trichoderma spirale | KT278911.1 | |
| | | 97.05 | 0.0 | Trichoderma spirale | KP263614.1 | |
| **45** | **1AG** | 95.82 | 0.0 | Bionectria sp. | HM849058.1 | NI |
| | | 95.80 | 0.0 | Clonostachys rosea | KJ619987.1 | |
| | | 95.80 | 0.0 | Bionectria ochroleuca | AY669327.1 | |
| | | 95.76 | 0.0 | Bionectria sp. | AB734437.1 | |
| **46** | **2X** | 97.79 | 0.0 | Roussoella solani | LC195220.1 | Rousseoella sp. |
| | | 97.79 | 0.0 | Roussoella solani | LC195219.1 | |
| | | 97.79 | 0.0 | Roussoella solani | LC195218.1 | |
| | | 97.78 | 0.0 | Neoroussoella leucaenae | NR_165226.1 | |
| | | 97.78 | 0.0 | Neoroussoella entadae | MK347786.1 | |

| | | | | | | |
|---|---|---|---|---|---|---|
| **47** | **1E** | 99.81 | 0.0 | Curvularia sp. | MG976352.1 | Curvularia sp. |
| | | 99.81 | 0.0 | Curvularia soli | NR_152503.1 | |
| | | 99.81 | 0.0 | Curvularia soli | KY905679.1 | |
| | | 99.81 | 0.0 | Curvularia sp. | KR093913.1 | |
| | | 99.81 | 0.0 | Curvularia senegalensis | HG779001.1 | |
| **48** | **1AU** | 100.00 | 0.0 | Fusarium camptoceras | KJ782411.1 | Fusarium sp. |
| | | 99.59 | 0.0 | Fusarium equiseti | AB425996.1 | |
| | | 99.59 | 0.0 | Fusarium oxysporum | JN400714.1 | |
| | | 99.59 | 0.0 | Fusarium sp. | HQ023205.1 | |
| | | 99.39 | 0.0 | Fusarium sp. | EF120420.1 | |
| **49** | **2I** | 99.82 | 0.0 | Mucor sp. | JF723592.1 | Mucor sp. |
| | | 99.65 | 0.0 | Mucoraceae sp. | KX148752.1 | |
| | | 99.33 | 0.0 | Mucor fragilis | FN650655.1 | |
| | | 98.64 | 0.0 | Mucor fragilis | KY047150.1 | |
| | | 98.64 | 0.0 | Mucor fragilis | KY047147.1 | |
| **50** | **2G** | 100.00 | 0.0 | Penicillium sp. | GU017521.1 | Penicillium sp. |
| | | 100.00 | 0.0 | Penicillium sp. | GU017519.1 | |
| | | 100.00 | 0.0 | Penicillium griseofulvum | FJ717687.1 | |
| | | 100.00 | 0.0 | Penicillium citrinum | MH864240.1 | |
| | | 100.00 | 0.0 | Penicillium citrinum | MH858380.1 | |
| **51** | **1AH** | 99.46 | 0.0 | Pleosporales sp. | KP306958.1 | NI |
| | | 99.29 | 0.0 | Roussoella solani | LC195220.1 | |
| | | 99.28 | 0.0 | Pleosporales sp. | KP263105.1 | |
| | | 99.28 | 0.0 | Ascomycota sp. | JX966643.1 | |
| | | 99.25 | 0.0 | Roussoella solani | MH923251.1 | |
| **52** | **1AY** | 97.89 | 0.0 | Myrothecium roridum | KJ815095.1 | Myrothecium sp. |
| | | 97.73 | 0.0 | Myrothecium roridum | KJ174523.1 | |
| | | 97.57 | 0.0 | Paramyrothecium roridum | MH856665.1 | |
| | | 97.57 | 0.0 | Paramyrothecium roridum | KU846301.1 | |
| | | 97.57 | 0.0 | Myrothecium roridum | JF343832.1 | |
| **53** | **1BG** | 95.16 | 0.0 | Myrothecium inundatum | KU856653.1 | Myrothecium sp. |
| | | 94.58 | 0.0 | Myrothecium inundatum | LC387258.1 | |
| | | 94.58 | 0.0 | Myrothecium inundatum | JN851023.1 | |
| **54** | **1R** | 97.93 | 0.0 | Colletotrichum dematium | AB046608.1 | Colletotrichum sp. |
| | | 97.93 | 0.0 | Colletotrichum capsici | EU056740.1 | |
| | | 97.76 | 0.0 | Colletotrichum dematium | AB042310.1 | |

| | | | | | | |
|---|---|---|---|---|---|---|
| | | 97.60 | 0.0 | Colletotrichum truncatum | KC460308.1 | |
| | | 97.60 | 0.0 | Colletotrichum truncatum | AJ301945.1 | |
| **55** | **1U** | 99.30 | 0.0 | Aspergillus tabacinus | LN898739.1 | Aspergillus sp. |
| | | 99.30 | 0.0 | Aspergillus sp. | FJ770064.1 | |
| | | 99.29 | 0.0 | Aspergillus versicolor | AM883155.1 | |
| | | 99.29 | 0.0 | Aspergillus tabacinus | LN898736.1 | |
| | | 99.28 | 0.0 | Aspergillus tabacinus | FR733851.1 | |
| **56** | **1W** | 97.59 | 0.0 | Colletotrichum dematium | AB046608.1 | Colletotrichum sp. |
| | | 97.59 | 0.0 | Colletotrichum capsici | EU056740.1 | |
| | | 97.42 | 0.0 | Colletotrichum dematium | Ab042310.1 | |
| | | 97.26 | 0.0 | Colletotrichum truncatum | MG677594.1 | |
| **57** | **1Z** | 99.35 | 0.0 | Nigrograna mackinnonii | NR_132037.1 | NI |
| | | 99.18 | 0.0 | Biatriospora mackinnonii | LN626660.1 | |
| | | 98.79 | 0.0 | Nigrograna mackinnonii | LN626659.1 | |
| | | 98.70 | 0.0 | Pleosporales sp. | KP263091.1 | |
| | | 98.70 | 0.0 | Nigrograna mackinnonii | KC288117.1 | |
| **58** | **2Q** | 99.28 | 0.0 | Ascomycota sp. | KP306958.1 | Roussoella sp. |
| | | 99.11 | 0.0 | Roussoella solani | LC195220.1 | |
| | | 99.11 | 0.0 | Roussoella solani | LC195219.1 | |
| | | 99.11 | 0.0 | Roussoella solani | LC195218.1 | |
| | | 99.11 | 0.0 | Pleosporales sp. | KP263105.1 | |
| **59** | **2V** | 98.79 | 0.0 | Fusarium chlamydo-sporum | MF072670.1 | Fusarium sp. |
| | | 98.42 | 0.0 | Fusarium chlamydo-sporum | AY213655.1 | |
| | | 98.42 | 0.0 | Fusarium equiseti | HQ649909.1 | |
| | | 98.41 | 0.0 | Fusarium equiseti | HQ649905.1 | |
| | | 98.23 | 0.0 | Fusarium incarnatum | MH857950.1 | |
| **60** | **2T** | 97.18 | 0.0 | Colletotrichum siamense | KP703406.1 | Colletotrichum sp. |
| | | 97.18 | 0.0 | Colletotrichum siamense | KP703404.1 | |
| | | 97.18 | 0.0 | Colletotrichum siamense | KP703403.1 | |
| | | 97.18 | 0.0 | Colletotrichum siamense | KP703402.1 | |
| | | 97.18 | 0.0 | Colletotrichum siamense | KP703385.1 | |
| **61** | **2AI** | 98.64 | 0.0 | Eutypella sp. | KY083415.1 | Eutypella sp. |
| | | 98.63 | 0.0 | Eutypella scoparia | JQ922162.1 | |
| | | 98.60 | 0.0 | Eutypella sp. | JQ411308.1 | |
| | | 98.44 | 0.0 | Peroneutypa scoparia | MG873478.1 | |
| | | 98.44 | 0.0 | Eutypella sp. | GQ293963.1 | |

| | | | | | | |
|---|---|---|---|---|---|---|
| **62** | **2AF** | 98.65 | 0.0 | Trichoderma asperellum | KC993073.1 | Trichoderma sp. |
| | | 98.64 | 0.0 | Trichoderma lieckfeldtiae | DQ109529.1 | |
| | | 98.64 | 0.0 | Trichoderma asperellum | KX510127.1 | |
| | | 98.65 | 0.0 | Trichoderma asperellum | MH153605.1 | |
| | | 98.65 | 0.0 | Trichoderma sp. | MK044000.1 | |
| **63** | **1O** | 99.19 | 0.0 | Aspergillus tabacinus | AM883155.1 | Aspergillus sp. |
| | | 99.00 | 0.0 | Aspergillus tabacinus | LN898739.1 | |
| | | 99.00 | 0.0 | Aspergillus tabacinus | LN898738.1 | |
| | | 99.00 | 0.0 | Aspergillus tabacinus | LN898737.1 | |
| | | 99.00 | 0.0 | Aspergillus tabacinus | FR733851.1 | |
| **64** | **1F** | 98.01 | 0.0 | Hypocrea sp. | HQ657316.1 | Trichoderma sp. |
| | | 98.01 | 0.0 | Trichoderma hamatum | MH864073.1 | |
| | | 98.01 | 0.0 | Trichoderma inhamatum | MH857107.1 | |
| | | 98.01 | 0.0 | Trichoderma harzianum | KT278882.1 | |
| | | 98.01 | 0.0 | Trichoderma harzianum | KR868358.1 | |
| **65** | **2D** | 99.38 | 00.0 | Penicillium sp. | KX148648.1 | Penicillium sp. |
| | | 99.17 | 0.0 | Penicillium sp. | GU017521.1 | |
| | | 99.17 | 0.0 | Penicillium sp. | GU017519.1 | |
| | | 99.17 | 0.0 | Penicillium griseofulvum | FJ717687.1 | |
| | | 99.17 | 0.0 | Penicillium citrinum | MH864240 | |
| **66** | **2AG** | 99.16 | 0.0 | Lasiodiplodia gonubiensis | KM006443.1 | Lasiodiplidia sp. |
| | | 99.16 | 0.0 | Lasiodiplodia gonubiensis | KF766191.1 | |
| | | 99.16 | 0.0 | Lasiodiplodia gonubiensis | NR_111218.1 | |
| | | 99.13 | 0.0 | Lasiodiplodia gonubiensis | AY639594.1 | |
| | | 98.95 | 0.0 | Lasiodiplodia gonubiensis | MH057180.1 | |
| **67** | **1BJ** | 99.08 | 0.0 | Trichoderma spirale | KT278877.1 | Trichoderma sp. |
| | | 99.08 | 0.0 | Trichoderma sp. | LC229678.1 | |
| | | 98.90 | 0.0 | Trichoderma spirale | KT278886.1 | |
| | | 98.90 | 0.0 | Trichoderma spirale | KR093830.1 | |
| | | 98.90 | 0.0 | Trichoderma spirale | EU280082.1 | |
| **68** | **2K** | 98.85 | 0.0 | Pestalotiopsis microspora | KT459350.1 | Pestalotiopsis sp. |
| | | 98.85 | 0.0 | Pestalotiopsis microspora | DQ456865.1 | |
| | | 98.85 | 0.0 | Pestalotiopsis microspora | AY924279.1 | |
| | | 98.85 | 0.0 | Pestalotiopsis sp. | HQ909077.1 | |
| | | 98.62 | 0.0 | Pestalotiopsis microspora | AY924271.1 | |

| | | | | | | |
|---|---|---|---|---|---|---|
| **69** | **1P** | 100.00 | 0.0 | Phoma sp. | JQ649376.1 | Phoma sp. |
| | | 100.00 | 0.0 | Phoma sp. | KP306990.1 | |
| | | 100.00 | 0.0 | Phoma sp. | KP306982.1 | |
| | | 100.00 | 0.0 | Phoma sp. | MN435151.1 | |
| | | 100.00 | 0.0 | Phoma sp. | KP306984.1 | |
| **70** | **1BD** | 96.79 | 0.0 | Xenomyrothecium tongaense | MH861297.1 | Myrothecium sp. |
| | | 96.43 | 0.0 | Myrothecium inundatum | KU856653.1 | |
| | | 96.22 | 0.0 | Myrothecium inundatum | LC387258.1 | |
| | | 96.22 | 0.0 | Myrothecium inundatum | LC387257.1 | |
| | | 96.22 | 0.0 | Myrothecium inundatum | LC387254.1 | |
| **71** | **1S** | 97.39 | 0.0 | Ramophialophora glo-bispora | KU746700.1 | NI |
| | | 96.91 | 0.0 | Ramophialophora glo-bispora | KU746699.1 | |
| | | 95.23 | 0.0 | Sordariomycetes sp. | KR909162.1 | |
| **72** | **1BM** | 99.60 | 0.0 | Penicillium sp. | GU017521.1 | Penicillium sp. |
| | | 99.60 | 0.0 | Penicillium griseofulvum | FJ717687.1 | |
| | | 99.60 | 0.0 | Penicillium citrinum | MH864240.1 | |
| | | 99.60 | 0.0 | Penicillium citrinum | MH858380.1 | |
| | | 99.60 | 0.0 | Penicillium citrinum | MH856132.1 | |
| **73** | **1BE** | 98.05 | 0.0 | Diaporthe sp. | MG976437.1 | Diaporthe sp. |
| | | 97.39 | 0.0 | Diaporthe sp. | MG976357.1 | |
| | | 97.05 | 0.0 | Diaporthe conorum | DQ116552.1 | |
| | | 96.59 | 0.0 | Diaporthe citri | KC343053.1 | |
| | | 96.59 | 0.0 | Diaporthe sp. | MG976377.1 | |
| **74** | **1BK** | 99.58 | 0.0 | Fusarium incarnatum | KU382560.1 | Fusarium sp. |
| | | 99.58 | 0.0 | Fusarium sp. | HQ023205.1 | |
| | | 98.97 | 0.0 | Fusarium oxysporum | JN400714.1 | |
| **75** | **1N** | 98.99 | 0.0 | Sarocladium glaucum | AB540577.1 | Sarocladium sp. |
| | | 98.57 | 0.0 | Sarocladium glaucum | NR_130686.1 | |
| | | 98.39 | 0.0 | Sarocladium glaucum | MH860868.1 | |
| | | 98.38 | 0.0 | Sarocladium glaucum | HG965020.1 | |
| | | 98.09 | 0.0 | Sarocladium glaucum | HG965019.1 | |
| **76** | **2AD** | 97.09 | 0.0 | Fusarium sp. | HQ023200.1 | Fusarium sp. |
| | | 96.42 | 0.0 | Fusarium robustum | NR_159851.1 | |
| | | 96.42 | 0.0 | Fusarium robustum | MH861018.1 | |
| | | 96.20 | 0.0 | Fusarium robustum | U85539.1 | |
| | | 95.75 | 0.0 | Fusarium sp. | U85548.1 | |

| | | | | | | |
|---|---|---|---|---|---|---|
| **77** | **2BC** | 99.79 | 0.0 | Bionectria sp. | KF746125.1 | NI |
| | | 99.57 | 0.0 | Clonostachys pityrodes | JQ411387.1 | |
| | | 99.17 | 0.0 | Bionectria pityrodes | AF210673.1 | |
| | | 98.75 | 0.0 | Bionectria pityrodes | AF210672.1 | |
| | | 98.63 | 0.0 | Clonostachys pityrodes | MH864280.1 | |
| **78** | **1BA** | 99.15 | 0.0 | Nigrospora pryzae | KJ159597.1 | Nigrospora sp. |
| | | 99.14 | 0.0 | Nigrospora sacchari-officinarum | NR_165926.1 | |
| | | 99.14 | 0.0 | Nigrospora sp. | MN215791.1 | |
| | | 98.85 | 0.0 | Nigrospora sp. | HQ630982.1 | |
| | | 98.74 | 0.0 | Nigrospora sp. | JN207335.1 | |
| **79** | **1AD** | 99.63 | 0.0 | Penicillium meleagrinum | HE962595.1 | Penicillium sp. |
| | | 99.28 | 0.0 | Penicillium meleagrinum | HM469412.1 | |
| | | 99.28 | 0.0 | Penicillium meleagrinum | EF198529.1 | |
| | | 99.28 | 0.0 | Penicillium meleagrinum | EF198530.1 | |
| | | 99.28 | 0.0 | Penicillium sp. | JF439496.1 | |
| **80** | **1D** | 92.90 | 9e-180 | Mucor nidicola | JF299215.1 | Mucor sp. |
| | | 92.86 | 0.0 | Mucor sp. | FJ210509.1 | |
| | | 90.02 | 0.0 | Mucor sp. | FJ210517.1 | |
| | | 89.95 | 0.0 | Mucor nidicola | HQ913647.1 | |
| **81** | **2BE** | 98.41 | 0.0 | Wiesneriomyces laurinus | KR822214.1 | Wiesneriomyces sp. |
| | | 87.27 | 2e-111 | Helicosporium gracile | AY916485.1 | |
| | | 86.87 | 1e-113 | Neofusiciccum mediterraneum | EU040221.1 | |

NI: Unable to get identification
